# The respiratory pressure—abdominal volume curve in a porcine model

**DOI:** 10.1186/s40635-017-0124-7

**Published:** 2017-02-28

**Authors:** Adrian Regli, Bart Leon De Keulenaer, Bhajan Singh, Lisen Emma Hockings, Bill Noffsinger, Peter Vernon van Heerden

**Affiliations:** 1 Intensive Care Unit, Fiona Stanley Hospital, 102-118 Murdoch Drive, Murdoch (Perth), WA 6150 Australia; 2 Intensive Care Unit, Sir Charles Gairdner Hospital, Hospital Avenue, Nedlands (Perth), WA 6009 Australia; 3 School of Medicine and Pharmacology, The University of Western Australia, Sterling Highway, Crawley (Perth), WA 6009 Australia; 4 Medical School, The Notre Dame University, Henry Road, Fremantle (Perth), WA 6959 Australia; 5 Department of Anaesthesia and Perioperative Medicine, The Alfred Hospital, Commercial Road, Prahran (Melbourne), VIC 3181 Australia; 6 Department of Pulmonary Physiology and Sleep Medicine, Sir Charles Gairdner Hospital, Hospital Avenue, Nedlands (Perth), WA 6009 Australia; 7 Faculty of Science, The University of Western Australia, Sterling Highway, Crawley (Perth), WA 6009 Australia; 8 West Australian Sleep Disorders Research Institute, QE II Medical Centre, Nedlands (Perth), WA 6009 Australia; 9 General Intensive Care Unit, Hadassah University Hospital, Kiryat Hadassah, Jerusalem, 91120 Israel

**Keywords:** Intra-abdominal pressure, Intra-abdominal hypertension, Abdominal compliance, Airway pressures, Respiratory compliance, Abdominal volume

## Abstract

**Background:**

Increasing intra-abdominal volume (IAV) can lead to intra-abdominal hypertension (IAH) or abdominal compartment syndrome. Both are associated with raised morbidity and mortality. IAH can increase airway pressures and impair ventilation. The relationship between increasing IAV and airway pressures is not known. We therefore assessed the effect of increasing IAV on airway and intra-abdominal pressures (IAP).

**Methods:**

Seven pigs (41.4 +/−8.5 kg) received standardized anesthesia and mechanical ventilation. A latex balloon inserted in the peritoneal cavity was inflated in 1-L increments until IAP exceeded 40 cmH_2_O. Peak airway pressure (pP_AW_), respiratory compliance, and IAP (bladder pressure) were measured. Abdominal compliance was calculated. Different equations were tested that best described the measured pressure-volume curves.

**Results:**

An exponential equation best described the measured pressure-volume curves. Raising IAV increased pP_AW_ and IAP in an exponential manner. Increases in IAP were associated with parallel increases in pP_AW_ with an approximate 40% transmission of IAP to pP_AW_. The higher the IAP, the greater IAV effected pP_AW_ and IAP.

**Conclusions:**

The exponential nature of the effect of IAV on pP_AW_ and IAP implies that, in the presence of high grades of IAH, small reductions in IAV can lead to significant reductions in airway and abdominal pressures. Conversely, in the presence of normal IAP levels, large increases in IAV may not affect airway and abdominal pressures.

**Electronic supplementary material:**

The online version of this article (doi:10.1186/s40635-017-0124-7) contains supplementary material, which is available to authorized users.

## Background

Intra-abdominal hypertension (IAH) is defined as a sustained intra-abdominal pressure (IAP) ≥ 12 mmHg [1]. IAH is common in critically ill patients [2] and is associated with an increased morbidity and mortality [3]. IAH is caused by additional intra-abdominal volume (IAV) within the confined abdominal cavity (e.g., retroperitoneal bleed, free fluid from massive fluid resuscitation, ascites, ileus with dilated bowel, etc.) or by reduced compliance of the abdominal wall (e.g., obesity or eschars in burns patients). If IAP is significantly increased or persists, an abdominal compartment syndrome may develop which is defined as a sustained IAP > 20 mmHg that is associated with new onset organ dysfunction [1]. Organ failure may include cardiac, respiratory, renal, and/or gastro-intestinal failure.

It has long been thought that a linear relationship exists between IAV and IAP [4–6]. However, in a recent review article, by extracting all available human IAV and IAP measurements from current literature, we were able to demonstrate an exponential relationship between IAV and IAP [7]. The exponential relationship between IAV and IAP is of interest because of the clinical consequences of IAH. Patients with IAH often have impaired lung function due to a cephaled displacement of the diaphragm, associated with impaired lung volumes and increased airway pressures resulting in difficulties in maintaining adequate ventilation [8].

Several therapeutic options exist to reduce IAP [9, 10]. These therapies are associated with small reductions in IAV and are therefore thought to have a small effect on IAP. However, due to the exponential relationship at higher IAP ranges, small reductions in IAV may indeed have significant effects on IAP.

The effect of changes in IAV on airway pressures is well known. We therefore aimed to characterize the influence of IAV on both IAP and airway pressures.

## Methods

Seven pigs were studied in a protocol to measure IAP and airway pressures caused by incremental increases in IAV. The Animal Ethics Committee of the University of Western Australia approved the study protocol (UWA RA/3/100/688). The study conformed to the regulations of the Australian code of practice for the care and use of animals for scientific purposes. Anesthesia, mechanical ventilation, surgical preparation, and instrumentation were performed as previously described [11] and are briefly outlined below.

### Animals

Seven Large White breed pigs [mean (SD) animal weight of 41.4 (+/−8.5) kg] received standardized anesthesia including initial sedation using intramuscular zolazepam/tiletamine (Zoletil ®) and xylazine followed by a combination of propofol, morphine, and ketamine for maintenance of anesthesia. At the end of the experimental protocol the pigs were euthanized with intravenous pentobarbitone. No neuromuscular blocking agents were used as they are infrequently used in our clinical practice and also to reduce the risk of awareness of pain in the animals. Adequacy of the depth of anesthesia was regularly assessed (lack of muscle tone, absence of spontaneous ventilatory effort).

### Mechanical ventilation and airway parameters

Mechanical ventilation (Servo 900, Siemens, Berlin, Germany) was maintained using constant tidal volumes of 8 mL/kg. Initial PEEP was 5 cmH_2_0. Respiratory rate was adjusted to maintain an end-tidal CO_2_ between 35 and 45 mmHg before the abdomen was inflated but not changed thereafter. Peak inspiratory pressure (pP_AW_) and dynamic respiratory system compliance (C_RS_) were obtained automatically from the ventilator. End-expiratory lung volume was measured at baseline IAP and PEEP of 5 cmH_2_O using the multiple breath nitrogen wash-out method as previously described [11]. Pressure-volume (P-V) curves were performed at PEEP 5 cmH_2_O and then at PEEP 15 cmH_2_O.

### Intra-abdominal pressure measurement

Urinary bladder pressure was used to assess IAP. For this, a 12F Foley catheter was placed in the urinary bladder via a caudal midline laparotomy and attached to a standard transducer system (Hospira, Lake Forest, IL). Mean pressures were measured from the mid-axillary line. Throughout the study, the animals remained in the supine position. A standardized injection volume of 25 mL of 0.9% NaCl (AbViser 300, Wolfe Tory Medical, Salt Lake City, UT) was used and 60 s relaxation time was allowed for before definitive measurement [1]. IAH was graded as recommended by the World Society of Abdominal Compartment Syndrome [1].

### Abdominal pressure-volume curve

A large intra-abdominal balloon (200 g latex weather balloon, Scientific Sales, Lawrenceville, NJ) was placed in the peritoneal cavity via midline laparotomy. Even placement of the balloon in the abdomen was ensured by visual inspection and partial inflation. A 1-L precision syringe (Vitalograph, Hamburg, Germany) was used to add air to the IAV in 1-L incremental steps. After each addition to IAV, we waited 10 s to allow pressures to equilibrate before assessing all parameters. Abdominal inflation was not continued beyond an IAP of 40.8 cmH_2_O (30.0 mmHg).

### Analysis and statistics

Absolute abdominal pressure-volume points were entered in a spreadsheet and analyzed using Excel (Microsoft, Redmond, WA, USA). All pressures were converted from millimeter of mercury into centimeter of water for better comparison between the IAP and pP_AW_ (1 mmHg = 1.3595 cmH_2_O). The change in IAV following addition of air to the intra-abdominal balloon was pressure-corrected using the Boyle equation (pressure-corrected additional IAV = measured additional × 1033/(1033 + IAP in centimeter of water), to compensate for the compressibility of air.

Two different equations were assessed for their accuracy at describing the IAP-IAV curve. First, the Venegas equation, *V* = *a* + [*b*/(1 + *e* ^−(*P*^^ − ^^*c*)/*d*^)], a logistic function was used. This has been used to describe the characteristic sigmoid shape of pulmonary [12] and other P-V curves [13]. In the original paper, *V* represents inflation or absolute lung volume, *P* represents airway opening or transpulmonary pressure, and *a*, *b*, *c*, and *d* represent fitting parameters. In the tested situation, *V* represented additional IAV, *P* represented absolute pressure (IAP or pPAW), and *a*, *b*, *c*, and *d* represent fitting parameters.

Second, an alternate exponential equation, *V* = *v* + *k* × Ln (*P*_0_ − *p*) where *V* represented additional IAV, *P*_0_ represented resting IAP (no additional IAV), and *v*, *k*, *p* represent fitting parameters, was tested. This equation has been used in the past to characterize lung elastic recoil and in other instances where pressures rise in near asymptotic fashion [14]. This exponential equation is single ended and exhibits near asymptotic shape at high volumes only whereas the Venegas equation exhibits true asymptotic shape at high and low volumes.

For each corresponding P-V data set, we used the Excel “Solver” function to find itting parameters that best described the measured P-V curve. The best fit was defined as a curve resulting in the smallest root mean square between the measured and calculated P-V points. Minimizing the residual sum of squares (RSS) is a standard method employed for fitting curves. The mean fitting parameter of all study subjects was used to plot a mean P-V curve.

Abdominal compliance (C_AB_) was defined as a measure of the ease of abdominal expansion, expressed as a change in intra-abdominal volume (IAV) per change in intra-abdominal pressure (IAP): AC = ΔIAV/ΔIAP [1]. C_AB_ is given as milliliter/centimeter of water for easier comparison with respiratory compliance.

Mann-Whitney rank sum test or Wilcoxon signed rank test was used as appropriate. A *p* value of <0.5 was considered statistically significant.

## Results

The subjects had a mean (SD) weight of 41.4 (+/−8.5) kg and end-expiratory lung volume of 1.68 (0.30) L. Baseline IAP was 5.0 cmH_2_O (3.7 mmHg). Expiratory tidal volume of 331 (68) mL and respiratory rate 34.1 (5.0) per minute were set at baseline (PEEP 5 cmH_2_O, no abdominal inflation). At 5 cmH_2_O PEEP, the highest applied IAP ranged from 42.1 to 55.7 cmH_2_O (31 to 41 mmHg), and additional IAV ranged from 7.7 to 14.3 L.

In comparison with the Venegas equation, the alternate exponential equation produced a P-V curve that better fitted the measured values (lower root mean square) (Additional file 1: Figure S1—P-V curves using Venegas and exponential equation, Additional file 2: Table S1—Equation parameters of intra-abdominal and airway pressure-volume curves).

**Fig. 1:**
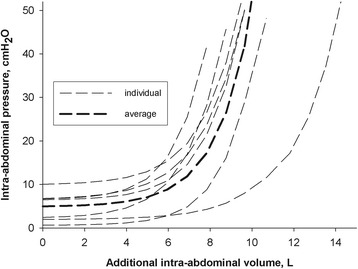
Pressure volume curves showing intra-abdominal pressure (IAP) in centimeter of water in function of increasing additional intra-abdominal volume in liters. An exponential equation was used to calculate IAP for individual animals (*narrow dashed curve*) and for the average of all animals (*bold dashed curve*)

We therefore subsequently used the alternate exponential equation. Figure 1 depicts P-V curves derived from the exponential equation of the average and of each individual of these animals. To calculate an expected IAP from a given additional IAV, the alternate exponential equation *V* = *v* + *k* × Ln (*P*_0_ − *p*) can be rearranged to *P*_0_ = *p* + Exp (*V* − *v*)/*k*.

After maximal abdominal inflation was achieved using on average 10.4 L (2.1) additional IAV, IAP after initial abdominal inflation at 5 cmH_2_O of positive end-expiratory pressure (PEEP) was higher than after subsequent abdominal re-inflation using the same additional IAV at 15 cmH_2_O of PEEP, 49.4 (4.1) vs 45.6 (2.3) cmH_2_O, respectively (*p* = 0.03) (Additional file 3: Figure S3—P-V curves at PEEP of 5 and 15 cmH_2_O). The abdominal compliance (C_AB_) at maximal IAV and IAP after initial (5 cmH_2_O PEEP) and repeat inflation (15 cmH_2_O PEEP) were 55.3 (5.0) mL/cmH_2_O and 62.0 (6.9) mL/cmH_2_O, respectively (*p* = 0.06).

**Fig. 2:**
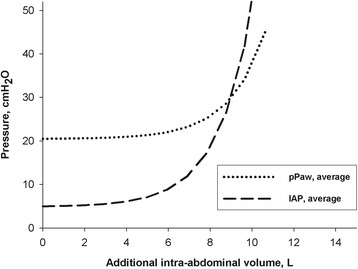
Pressure volume curves showing intra-abdominal pressure (IAP) (*dashed curve*) and peak airway pressure (pP_AW_) (*dotted curve*) in centimeter of water in function of increasing additional intra-abdominal volume in liters. An exponential equation was used to calculate IAP and pP_AW_ for the average of all animals

**Fig. 3:**
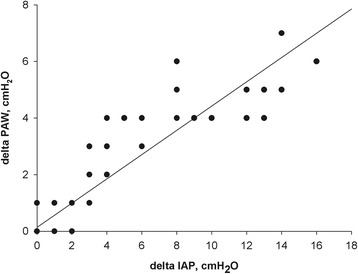
Delta peak airway pressure (pP_AW_) in centimeter of water as a function of delta IAP in centimeter of water. Delta pP_AW_ = 0.14 + 0.43 × delta IAP, *R* ^2^ = 0.83, *p* < 0.001

**Fig. 4:**
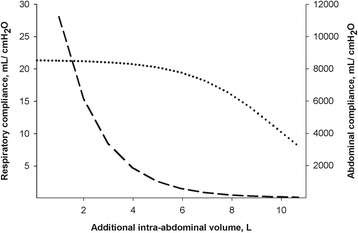
Average abdominal compliance in milliliter per centimeter of water (*dashed curve*) and dynamic respiratory system compliance in milliliter per centimeter of water (*dotted curve*) as a function of increasing additional intra-abdominal volume in liters. Abdominal compliance was calculated from the difference of additional intra-abdominal volume per difference of resulting intra-abdominal pressure. Dynamic respiratory compliance was taken from the ventilator

With an increasing amount of additional IAV, IAP and to a lesser extent pP_AW_ increased exponentially (Fig. 2). There was a directly proportional relationship between delta pP_AW_ as a function of delta IAP, with a strong correlation (delta pP_AW_ = 0.14 + 0.43 × delta IAP, *R* ^2^ = 0.83, *p* < 0.001). Hence, abdomino-thoracic transmission approximated 40% (Fig. 3). With increasing IAP, C_AB_ and C_RS_ decreased (Fig. 4).

We calculated the effect of an additional IAV of 500 mL on pP_AW_ and IAP (Table 1). The addition of 500 mL IAV increased pP_AW_ and IAP to a greater extent at higher grades of IAH than at lower grades of IAH (i.e., reduced compliance at higher levels of IAH).

**Table 1:** Effect of additional 500 mL intra-abdominal volume on intra-abdominal and peak airway pressure at different grades of intra-abdominal hypertension

Given IAP, cmH_2_O (mmHg)	IAH grade	P_AW_ at given IAP, cmH_2_O	Additional IAV at given IAP, L	IAV increased by 0.5 L, L	IAP after addition of 0.5 L IAV, cmH_2_O (mmHg)	Resulting increase in IAP, cmH_2_O (mmHg)	C_AB_, mL/mmHg	Resulting increase in P_AW_, cmH_2_O
5.0 (3.7)	Baseline	20.5	0.0	0.5	5.0 (3.7)	0.0 (0.0)	–	0.0
16.3 (12.0)	1	24.8	7.7	8.2	20.4 (15.0)	4.0 (3.0)	168	1.5
21.8 (16.0)	2	26.8	8.4	8.9	27.7 (20.4)	6.0 (4.4)	114	2.2
28.5 (21.0)	3	29.3	8.9	9.4	36.9 (27.2)	8.4 (6.2)	81	3.0
35.3 (26.0)	4	31.8	9.3	9.8	46.1 (33.9)	10.8 (7.9)	63	3.9

Values are given in mean (SD). Pigs weight was 41.4 (8.5) kg. *IAP* intra-abdominal pressure, *IAH* intra-abdominal hypertension, grading of IAH according to the World Society of Abdominal Compartment Syndrome [1], P_AW_ peak airway pressure, *IAV* intra-abdominal volume, *C*_*AB*_ abdominal compliance

## Discussion

The main findings in this animal model were that (a) raising IAV increased pP_AW_ and IAP in an exponential manner, (b) raising IAV decreased C_AB_ and C_RS_, and (c) there was approximately a 40% transmission of IAP to pP_AW_.

### IAV increased pP_AW_ and IAP in an exponential manner

We aimed to characterize how abdominal volumes influenced airway pressures and IAP. The IAV that produced an IAP >40 cmH_2_O (30 mmHg) varied between subjects. We therefore explored functions that could describe generic P-V curves of all the animals studied.

Functions have the advantage of allowing extrapolations even in the setting of a non-linear curve if a certain number of pressure-volume (P-V) values are known. We first tested the Venegas equation for the additional IAV—pP_AW_ and IAP relationship frequently used to describe a respiratory P-V curve [12]. The Venegas equation has been used to describe P-V curves other than respiratory [13]. We found that our alternative exponential function characterized the changes in pP_AW_ and IAP with additional IAV more accurately than the Venegas equation. Exponential equations have been used to describe respiratory P-V curves [15].

An exponential relationship between IAV and IAP has been found in a previous animal study [16]. In a recent review article, we extracted all available human IAV and IAP measurements from the current literature [7]. In contrast to previous studies, we analyzed multiple IAV and IAP measurements that included multiple measurements and/or were derived from a larger IAP range (greater than 15 mmHg, the upper limit during laparoscopy). We found an exponential relationship between IAV and IAP. The pseudo-linear relationship between IAV and IAP found in previous studies can be explained by the relatively low IAP range and/or small number of measurements examined [4–6]. To our knowledge, we are the first to report an exponential relationship between IAV and pP_AW_.

### Abdominal compliance

After completion of the first P-V curves at 5 cmH_2_O PEEP, we performed a second set of P-V curves at 15 cmH_2_O PEEP levels. The higher C_AB_ at 15 cmH_2_O of PEEP indicates a left shift of the P-V curves. This was opposite to what we anticipated and suggests that a considerable amount of “pre-stretching” occurred during the initial abdominal inflation rather than being a result of PEEP itself. We therefore only presented data obtained at PEEP of 5 cmH_2_O. Data from the literature suggests that stretching of the abdominal wall can lead to long-term changes in the elastic properties of the abdominal wall thereby improving abdominal compliance [7]. Unfortunately, we did not perform a third P-V curve at 5 cmH_2_O PEEP following the P-V curve at 15 cmH_2_O PEEP to confirm our hypothesis of the occurrence of pre-stretching.

Conceptually, three phases of abdominal pressure-volume behavior exist that occurs to some degree in parallel: the initial reshaping phase (minimal change in IAP despite large IAV change), the subsequent stretching phase, and finally, the pressurization phase (large IAP changes as a result of small IAV changes) [7]. Pre-stretching regularly occurs as an adaptive response to a chronic disease process (e.g., growing ascites or pregnancy), but it has also been shown to occur in the acute setting within a short period of time (e.g., during laparoscopy) [7].

The WSACS (www.wsacs.org) defines abdominal compliance as a measure of the ease of abdominal expansion, determined by the elasticity of the abdominal wall and diaphragm and expressed as a change in IAV per change in IAP (L/mmHg) [1, 17, 18]. Not surprisingly additional IAV decreased C_AB_ and C_RS_.

### Abdomino-thoracic transmission

We found approximately 40% of abdomino-thoracic transmission, whereby pP_AW_ increased due to raising IAP. There is a paucity of literature examining the effect of various IAP on airway pressures. When averaging the results of three porcine studies, we found an approximate 40% abdomino-thoracic transmission [11, 19, 20]. In keeping with this, Cortes-Puentes and colleges found an approximate 50% abdomino-thoracic transmission in pigs [21]. These results should be treated with caution as abdomino-thoracic transmission is likely to be different in critically ill patients. Factors such as obesity, presence of pleural effusions, and lung compliance may well substantially influence abdomino-thoracic transmission [22]. We could only locate one study in human subjects from which abdomino-thoracic transmission can be derived. Torquato et al. placed 5 kg weights on the abdomen of mechanically ventilated, critically ill patients. The average IAP increased from 10.5 to 15.6 cmH_2_O and plateau airway pressures from 22.4 to 23.6 cmH_2_O equating to an approximate 20% abdomino-thoracic transmission [23].

### Thoraco-abdominal transmission

Thoraco-abdominal transmission can be explored by assessing the effect of either different PEEP levels or different tidal volumes on IAP. We attempted to examine the influence of PEEP on IAP. However, as we did not randomize the levels of PEEP and as described above, we believe that the unexpected findings may be the result of pre-stretching rather than the effect of different levels of PEEP. Therefore, we were unable to examine the influence of PEEP on thoraco-abdominal P-V curves.

Published reports suggest that PEEP has either no or minimal effect on IAP in animals and in humans [24]. We found in an animal experiment that PEEP did not influence IAP [8]. In humans, PEEP appears to increase IAP and the calculated thoraco-abdominal transmission ranges between 0.2 and 0.4 cmH_2_O increase in IAP for each centimeter of water of PEEP [23, 25, 26]. Other studies have found tidal volume to have a significant impact on IAP [15].

The thoraco-abdominal transmission has been suggested to provide an estimate of the C_AB_ by measuring the influence of different tidal volumes on the changes in IAP [18].

### How is this study useful?

It is important for the critical care physician to be aware of the exponential nature of respiratory and abdominal pressure—IAV curve. An exponential pressure-volume relationship is the well-known Monro-Kellie doctrine applied in patients with intra-cranial hypertension [27].

At the lower IAV spectrum (i.e., patients with normal IAP), the respiratory and abdominal pressure—IAV curve is flat. This means that the abdominal cavity can accommodate several liters of additional IAV (i.e., intra- or retro-peritoneal hemorrhage) with little effect on airway or abdominal pressures. In using our pigs of around 40 kg as examples, an additional IAV of 4 L increased IAP by only 0.7 cmH_2_O (0.5 mmHg) and the effect on airway pressures was negligible. Therefore, an absent rise in IAP does not exclude an intra-abdominal or retroperitoneal hemorrhage in the lower IAV spectrum.

In the high IAV spectrum (i.e., patients with IAH), the respiratory and abdominal pressure—IAV curve is steep. Small changes in IAV can significantly affect airway pressure and IAP. Therefore, it is important to measure IAP regularly in patients at risk of developing IAH. Especially in patients with impending ACS, a small increase in IAV can easily progress to an ACS.

There is a high incidence of IAH in patients with respiratory failure [28]. IAH contributes to morbidity and mortality in patients with acute respiratory distress syndrome (ARDS) [15, 25, 29, 30]. Airway pressures are often high when ventilating patients with ARDS, and it has been suggested that plateau pressure should be limited to 30 cmH_2_O [31]. These recommendations do not take IAP into account even though IAH has been shown to increase airway pressures in this current study and in previous animal and human studies [8, 32]. At least in patients with ARDS, recent studies suggest it is more important to limit the driving airway pressure than it is to limit plateau airway pressure [33]. Of note is that a sudden rise in airway pressures may reflect an acute increase in IAV and should prompt an abdominal examination to exclude an intra-abdominal pathology.

When aiming to reduce airway pressures and/or IAP in patients with IAH/abdominal compartment syndrome, it is useful to understand that small reductions in IAV can significantly improve airway pressure and IAP. In example, in this study, at grade IV IAH, a 500-mL reduction in IAV reduced pP_AW_ by 4 cmH_2_O and IAP by 11 cmH_2_O (8 mmHg). This observation is similar to the applied Monro-Kellie principle where drainage of small amount of cerebral spinal fluid can significantly reduce intra-cranial pressure in patients with intra-cranial hypertension [27].

There are multiple methods of reducing IAV, and the best management depends largely on the etiology of IAH [1]. Apart from diuresis (e.g., furosemide), renal replacement therapy, and surgery (e.g., removal of hematoma or decompressive laparotomy), percutaneous drainage of peritoneal fluid has been shown to be equally effective in reducing IAP [34–36]. In a case series, Reed et al. present 12 patients in which percutaneous drains were inserted in patients with IAH [35]. In the patients with higher pre-drainage IAP of smaller amount fluid removal led to greater decreases in IAP than in patients with smaller pre-drainage IAP.

### Limitations

This study has several limitations: (a) These findings have been obtained in an animal model, limiting the transfer of our results into clinical practice. (b) We used a healthy lung model but critically ill patients frequently have injured lungs with reduced lung compliance. (c) This model assessed the effect of increasing IAV but not that of decreasing abdominal wall compliance on pP_AW_ and IAP. Although decreased abdominal wall compliance does occur, increased IAV is the more dominant process in critically ill patients [7]. The abdominal closure may have decreased abdominal wall compliance [7]. (d) In clinical practice IAH arises more often on the basis of excess in intra-abdominal fluid than of an excess in intra-abdominal gas. We used air to increase IAV but corrected the additional IAV to account for the compressibility of gas under pressure using the Boyle’s equation. Although we visually ensured even distribution of the abdominal balloon we cannot exclude potential asymmetrical IAP distribution. (e) We measured mean IAP and not end-expiratory IAP as recommended by the WSACS [1]. (f) We measured dynamic respiratory compliance and peak airway pressure and not plateau airway pressure and static respiratory compliance. We assume that the same principles apply for plateau pressure. In a previous animal experiment [19], we found that plateau pressure paralleled peak airway pressure (data not published). (g) We did not observe any spontaneous diaphragmatic activity. However, we cannot totally rule out diaphragmatic activity potentially influencing our results as we did not use neuromuscular blocking agents. (h) We did not perform a third P-V curve at 5 cmH_2_O PEEP following the P-V curve at 15 cmH_2_O PEEP to confirm our hypothesis that pre-stretching had occurred.

## Conclusions

In conclusion, in an animal model, we found that raising IAV increased pP_AW_ and IAP in an exponential manner. The exponential nature of IAV on pP_AW_ and IAP suggests that the effect of a given change in IAV on pP_AW_ and IAP will be greater at high than at low levels of IAP. In other words, in subjects with normal IAP, large increases in IAV will not affect airway pressure or IAP. But at high grades of IAH, small reductions in IAV can significantly improve airway and abdominal pressures.

## Additional files

Additional file 1: Figure S1. Intra-abdominal pressure (IAP) in centimeter of water in function of increasing additional intra-abdominal volume (IAV) in liters. Example of one pig showing measured IAP values (crosses), calculated IAP values using Venegas equation (dotted curve) and exponential equation (dashed curve). Venegas equation: *V* = *a* + [*b*/(1 + *e* ^−(*P* − *c*)/*d*^)] [12], V represents additional IAV, P represents absolute IAP, and *a*, *b*, *c*, and *d* represents fitting parameters. Exponential equation: *V* = *v* + *k* × Ln (*P* − *p*) where *V* represents additional IAV, *P* represents absolute IAP, and *v*, *k*, *p* represents fitting parameters. (TIF 384 kb)
Additional file 2: Table S1. Equation parameters of intra-abdominal and airway pressure-volume curves. (DOCX 55 kb)
Additional file 3: Figure S3. Pressure-volume curves showing intra-abdominal pressure in centimeter of water at the initial PEEP level of 5 cmH_2_O (dashed curve) and the subsequent PEEP level of 15 cmH_2_O (solid curve) in function of increasing additional intra-abdominal volume in liters. (TIF 445 kb)

## Abbreviations

C_AB_: Abdominal compliance
C_RS_: Dynamic respiratory system compliance
IAH: Intra-abdominal hypertension
IAP: Intra-abdominal pressure
IAV: Intra-abdominal volume
PEEP: Positive end-expiratory pressure
pP_AW_: Peak airway pressure
P-V: Pressure-volume
