# Supplementary figures and images for: The respiratory pressure—abdominal volume curve in a porcine model
Source: Intensive Care Med Exp. 2017 Feb 28;5:11. doi: 10.1186/s40635-017-0124-7 (PMC5328886; doi:10.1186/s40635-017-0124-7)

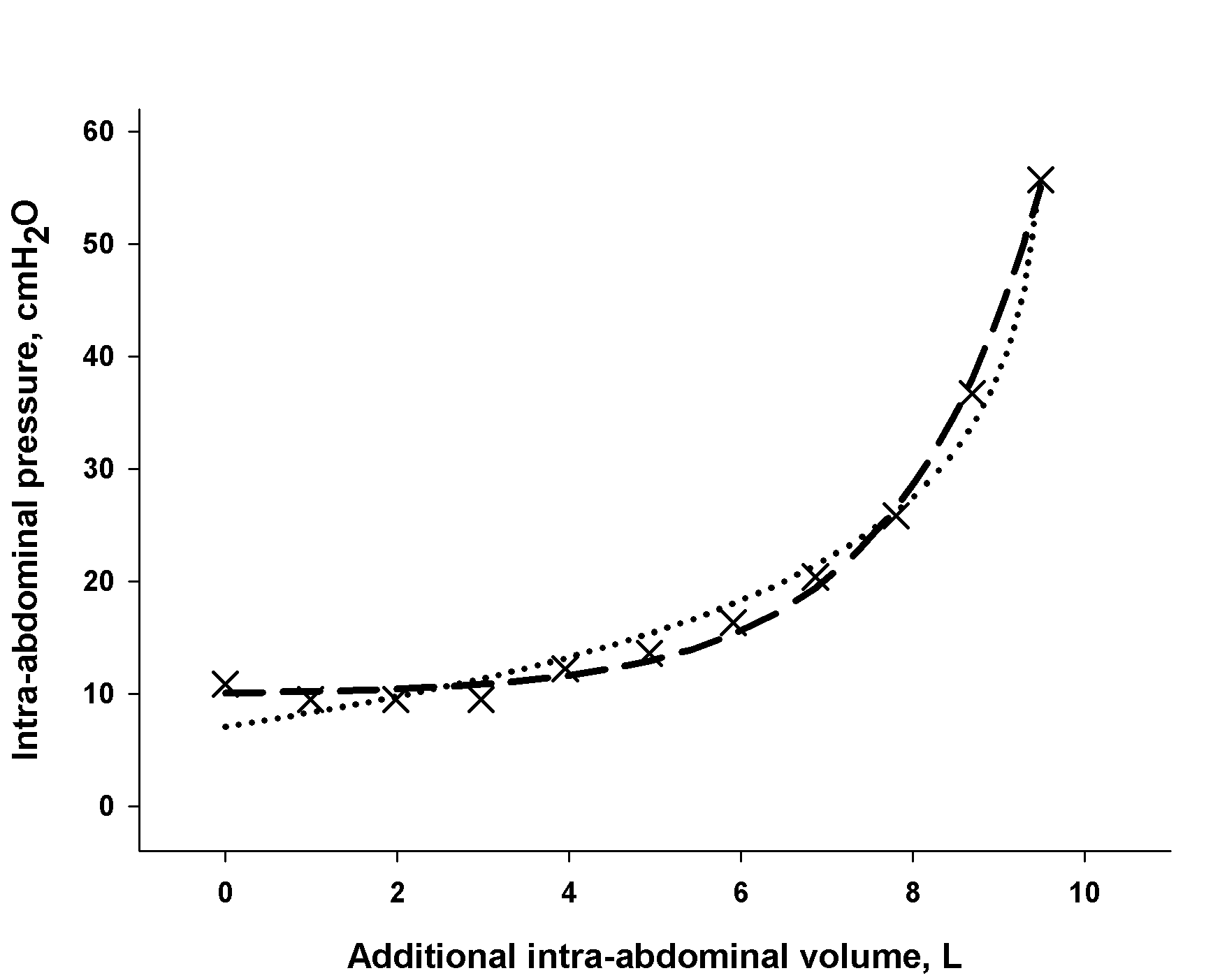

Supplement: Additional file 1: Figure S1. — Intra-abdominal pressure (IAP) in centimeter of water in function of increasing additional intra-abdominal volume (IAV) in liters. Example of one pig showing measured IAP values (crosses), calculated IAP values using Venegas equation (dotted curve) and exponential equation (dashed curve). Venegas equation: V = a + [b/(1 + e −(P − c)/d)] [12], V represents additional IAV, P represents absolute IAP, and a, b, c, and d represents fitting parameters. Exponential equation: V = v + k × Ln (P − p) where V represents additional IAV, P represents absolute IAP, and v, k, p represents fitting parameters. (TIF 384 kb) [file 40635_2017_124_MOESM1_ESM.tif]

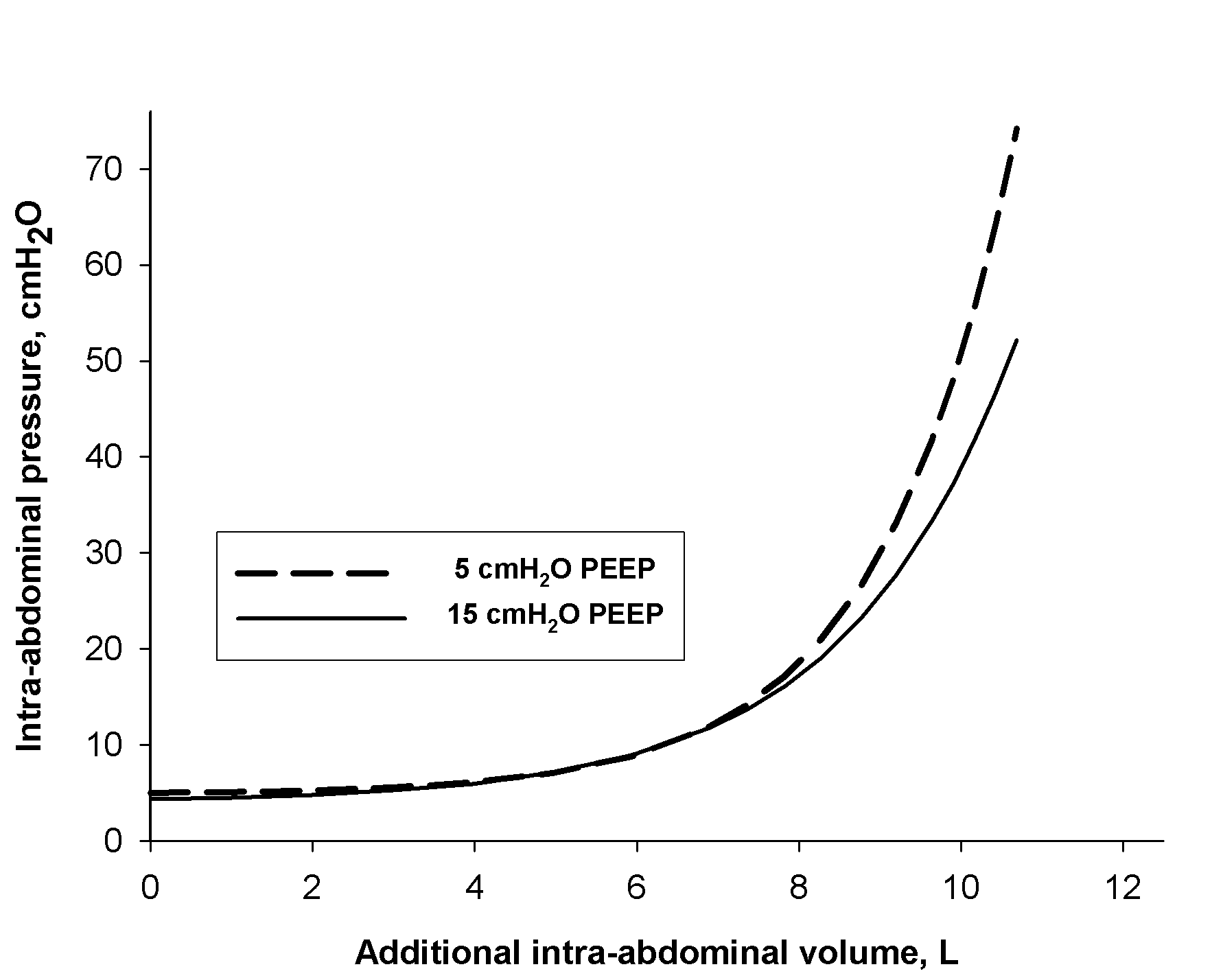

Supplement: Additional file 3: Figure S3. — Pressure-volume curves showing intra-abdominal pressure in centimeter of water at the initial PEEP level of 5 cmH2O (dashed curve) and the subsequent PEEP level of 15 cmH2O (solid curve) in function of increasing additional intra-abdominal volume in liters. (TIF 445 kb) [file 40635_2017_124_MOESM3_ESM.tif]
