# Supplementary material for: The respiratory pressure—abdominal volume curve in a porcine model
Source: Intensive Care Med Exp. 2017 Feb 28;5:11. doi: 10.1186/s40635-017-0124-7 (PMC5328886; doi:10.1186/s40635-017-0124-7)
Supplement: Additional file 2: Table S1. — Equation parameters of intra-abdominal and airway pressure-volume curves. (DOCX 55 kb) [file 40635_2017_124_MOESM2_ESM.docx]

**Supplemental Digital Content 2 - Table - Equation parameters of intra-abdominal and airway pressure-volume curves**

|  | Venegas |  |  |  |  | Alternate Exponential |  |  |  | p-value |
| --- | --- | --- | --- | --- | --- | --- | --- | --- | --- | --- |
|  | a | b | c | d | RMS | p | v | k | RMS |  |
| IAP | 10.1 (1.9) | -98000 (61349) | -86.0 (26.3) | -10.8 (1.1) | 2.0 (0.3) | 4.9 (3.1) | 3.7 (1.3) | 1.7 (0.2) | 0.9 (0.2) | 0.01 |
| pP_AW_ | 10.2 (2.0) | -116985 (55261) | -18.6 (12.5) | -4.2 (1.3) | 1.1 (0.4) | 20.5 (3.1) | 5.2 (1.3) | 1.7 (0.4) | 0.4 (0.1) | 0.02 |

Venegas equation = V = a + [b/(1+e ^–(P-d)/d^)]; Alternate exponential equation, P_0_ = p + k *exp (V-v); RMS, root mean square; AP, intra-abdominal pressure; pP_AW_, peak airway pressure; Mann-Whitney Rank Sum test was used to calculate p value between the two RMS.
